# Viable cryopreserved human bone graft exhibit superior osteogenic properties in mandibular lateral augmentation

**DOI:** 10.1038/s41598-023-28170-6

**Published:** 2023-01-25

**Authors:** Daniel Deluiz, Gaëtan J.-R. Delcroix, Samira R. G. Fraga, Gianluca D’Ippolito, Cristina Grau-Monge, Andrea Bonnin-Marquez, Teresita Reiner, Thaís Amadeu, Eduardo M. B. Tinoco, Paul Christian Schiller

**Affiliations:** 1 Department of Periodontology, State University of Rio de Janeiro, Boulevard 28 de Setembro, 157 - 2º andar - sala 10, Rio de Janeiro, RJ CEP 20551-030 Brazil; 2 Department of Orthopedics, University of Miami, Miami, FL USA; 3 College of Allopathic Medicine, Nova Southeastern University, Fort Lauderdale, FL USA; 4 Geriatric Research Education and Clinical Center, Miami VA Healthcare System, Miami, FL USA; 5 Department of Biomedical Engineering, College of Engineering, University of Miami, Miami, FL USA; 6 Department of Pathology and Laboratories, State University of Rio de Janeiro, Rio de Janeiro, RJ Brazil

**Keywords:** Stem-cell research, Preclinical research

## Abstract

Lack of bone volume to place dental implants is frequently a problem in the reconstruction of edentulous patients. Even though autografts are the gold standard for jaw regeneration, morbidity associated with the harvesting site stimulates the demand for other substitutes. The aim of this study is to characterize the incorporation and the osteogenic ability of a viable cryopreserved human bone graft (VC-HBG) in the mandibular augmentation in rats. Bone chips from fresh human vertebrae cadaveric donors were processed, cryoprotected and deep-frozen at − 80 °C maintaining its cell viability. A jaw augmentation model was used in 20 athymic nude rats allocated into 2 groups to either receive the VC-HBG or an acellular graft as control (A-HBG). The assessment of the grafts' incorporation was performed at 4 and 8 weeks by micro-CT, histomorphometry and immunohistochemistry. Bone volume gain was significantly higher for the VC-HBG group at both time points. At 4 weeks, the A-HBG group presented significantly higher mineral density, but at 8 weeks, the VC-HBG group showed significantly higher values than the A-HBG. There was no statistical difference between VC-HBG and A-HBG groups at 4-weeks for remaining graft particles, while at 8 weeks, the VC-HBG group showed significantly less graft remnants. Collagen I, osteopontin and tartrate-resistant acid phosphatase expression were significantly higher in the VC-HBG group at both time points, while osteocalcin expression was significantly higher in the VC-HBG group at 8-weeks compared to the A-HBG group. This experimental research demonstrated that the VC-HBG shows positive osteogenic properties, greater bone formation, higher rate of bone remodeling and a better overall incorporation in rats' mandibles compared to the A-HBG.

## Introduction

Bone atrophy is a physiological sequel after tooth loss and commonly results in insufficient structure to stabilize dental implants, leading to higher failure rates, unsatisfactory aesthetic results or poor prosthetic design. In order to adequately reconstruct bone volume and alveolar ridge anatomy prior to implant placement, several surgical techniques and biomaterials are currently available to the clinician^1,2^. Autologous grafts have been extensively investigated and the scientific literature reports favorable results with good success rates, achieving effective and stable volume gain with no concern regarding immune response or disease transmission. Therefore, autografts are considered the gold standard material for bone reconstruction in the dental field^3,4^. However, morbidity related to the donor surgical harvest site and risk of complications such as paresthesia, hemorrhage, damage to vital structures and excessive edema have encouraged the researchers to seek the development of grafting biomaterials that are able to successfully replace the autogenous bone during alveolar ridge augmentation procedures^5–7^.

Osteogenic properties of autografts are due to their unique content in viable cells and inductive proteins. Tissue engineering triad is achieved with cells, chemical signaling and a proper scaffold that will allow cells to migrate, adhere and produce new tissue^1^. Bone marrow-derived stromal cells (MSCs) have the potential to differentiate into distinct cell lineages, including bone-forming osteoblasts. The attempts of improving bone grafts using MSCs have shown greater and faster bone volume formation compared to commonly used biomaterial grafts^8,9^. In a recent study by our research group, it was observed that human cadaveric bone microparticles seeded with MSCs isolated from bone marrow showed positive osteogenic properties, resulting in significantly faster bone formation along with a higher bone turnover rate and better overall incorporation, when compared to their acellular counterpart^10^. More recently, a new human bone processing method has allowed the production of a bone grafting material preserving its viable cell content during cryopreservation.

The aim of this study is to characterize the incorporation and determine the osteogenic ability of this novel viable cryopreserved human bone graft (VC-HBG) during mandibular lateral augmentation in rats.

## Results

### Surgical procedure

The surgical procedure used is described in greater details in the material and “Methods” section. All animals tolerated the surgeries well and no adverse events were observed post-operatively. Signs of infections that could compromise histological, immunohistochemical and tomographic analyzes were also absent from all animals.

### Micro-CT evaluation

The quantitative variables analyzed by micro-CT are presented through mean, median, standard deviation, minimum, maximum and interquartile range by type of graft on Table 1.

**Table 1:** Descriptive analysis of tomographic quantitative variables by type of graft.

	Volume (mm^3^)		Bone mineral density		Percent bone volume		Bone surface/volume ratio		Bone surface density	
	A-HBG	VC-HBG	A-HBG	VC-HBG	A-HBG	VC-HBG	A-HBG	VC-HBG	A-HBG	VC-HBG
n	10	10	10	10	10	10	10	10	10	10
Mean	23.80	41.78	0.64	0.63	49.14	41.71	23.60	25.40	10.42	10.47
Median	21.26	41.51	0.65	0.62	47.21	41.84	25.46	24.85	10.57	10.60
Standard deviation	12.55	8.34	0.08	0.04	16.32	6.39	7.04	3.02	0.78	1.11
Minimum	8.37	27.39	0.55	0.58	25.31	32.34	13.64	21.64	8.59	8.42
Maximum	39.85	53.31	0.77	0.72	77.50	50.09	33.94	30.53	11.48	12.17
Interquartile range	23.28	13.19	0.14	0.07	24.57	12.90	11.95	5.41	0.67	1.73

*A-HBG* acellular-human bone graft, *VC-HBG* viable cryopreserved-human bone graft.

#### Bone volume (BV) gain

**Figure 1:**
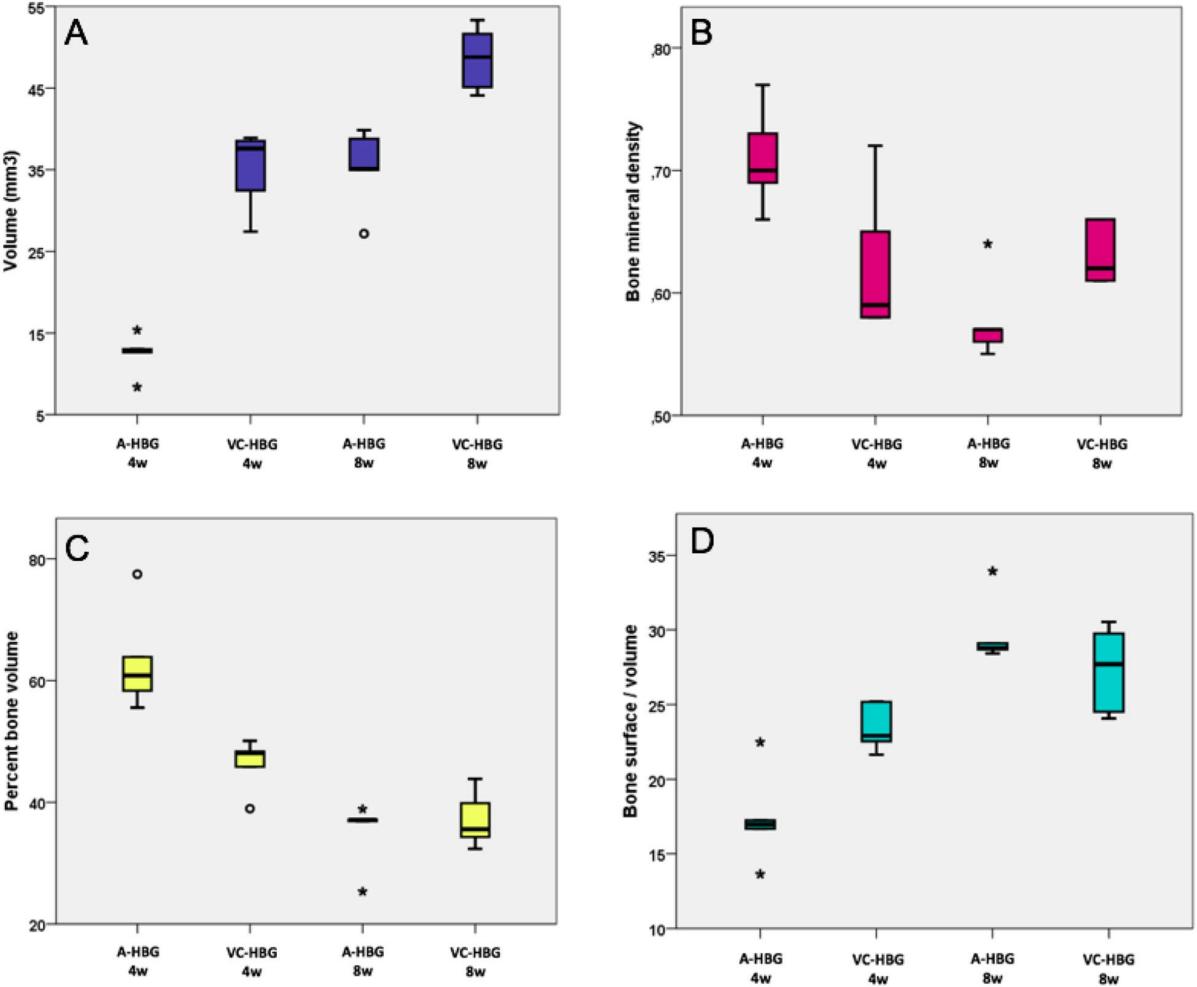
Association between bone volume gain (mm^3^) (**A**), bone mineral density (BMD) (**B**), percentage of bone volume (**C**), bone surface/total volume ratio (**D**) and graft type at each time point. *A-HBG* acellular-human bone graft, *VC-HBG* viable cryopreserved-human bone graft.

At 4 weeks, the average BV in the VC-HBG was significantly higher (34.97 ± 4.97 mm^3^) than the mean of the A-HBG group (12.43 ± 2.53 mm^3^, *p* < 0.001). At 8 weeks, there was also a statistically significant difference (*p* = 0.002) between the mean BV in the VC-HBG (48.59 ± 3.99 mm^3^) and A-HBG group (35.18 ± 4.98 mm^3^), see Fig. 1A. VC-HBG presented significantly greater volume than A-HBG grafts at 4-week and 8-week time points (*p* < 0.001).

#### Bone mineral density

At 4 weeks, the A-HBG group presented significantly higher mineral density (0.71 ± 0.04 mg/cm^3^) than the VC-HBG group (mean = 0.62 ± 0.06 mg/cm^3^, *p* = 0.032). Conversely, at 8-weeks, the group that received VC-HBG (mean: 0.63 ± 0.03 mg/cm^3^) had significantly higher values (*p* = 0.025) than the A-HBG group (mean: 0.58 ± 0.04 mg/cm^3^), see Fig. 1B.

#### Percent bone volume (PBV)

At 4 weeks, the mean PBV of the A-HBG group was higher than the VC-HBG group (63.22 ± 8.55% and 46.25 ± 4.36%, respectively), *p* = 0.004. However, at 8 weeks, the A-HBG and VC-HBG groups showed no statistically significant difference (*p* > 0.05), see Fig. 1C. VC-HBG grafts at 8 weeks showed a significantly lower PBV than A-HBG at 4 weeks (*p* = 0.004).

#### Bone surface/volume ratio (BS/VR)

BS/VR were significantly higher for the VC-HBG group (23.49 ± 1.61) compared to the A-HBG group (17.41 ± 3.19) at the 4-weeks time point, as shown in Fig. 1D (*p* = 0.005).

### Histomorphometric analyses

Descriptive quantitative histomorphometric variables are presented through mean, median, standard deviation, minimum, maximum and interquartile range by type of graft on Table 2.

**Table 2:** Descriptive analysis of quantitative histomorphometric variables by type of graft.

	New bone		Graft Remains		Calcified tissue	
	A-HBG (%)	VC-HBG (%)	A-HBG (%)	VC-HBG (%)	A-HBG (%)	VC-HBG (%)
n	10	10	10	10	10	10
Mean	13.49	45.44	34.24	24.98	48.43	50.52
Median	11.88	46.28	35.36	22.55	44.31	50.54
Standard deviation	11.67	6.55	17.74	19.41	13.10	5.88
Minimum	1.82	36.40	8.37	3.22	35.35	37.95
Maximum	34.25	55.00	64.66	59.02	73.38	57.02
Interquartile range	20.52	11.78	30.63	34.52	22.39	8.45

*A-HBG* acellular-human bone graft, *VC-HBG* viable cryopreserved-human bone graft.

#### New bone formation

**Figure 2:**
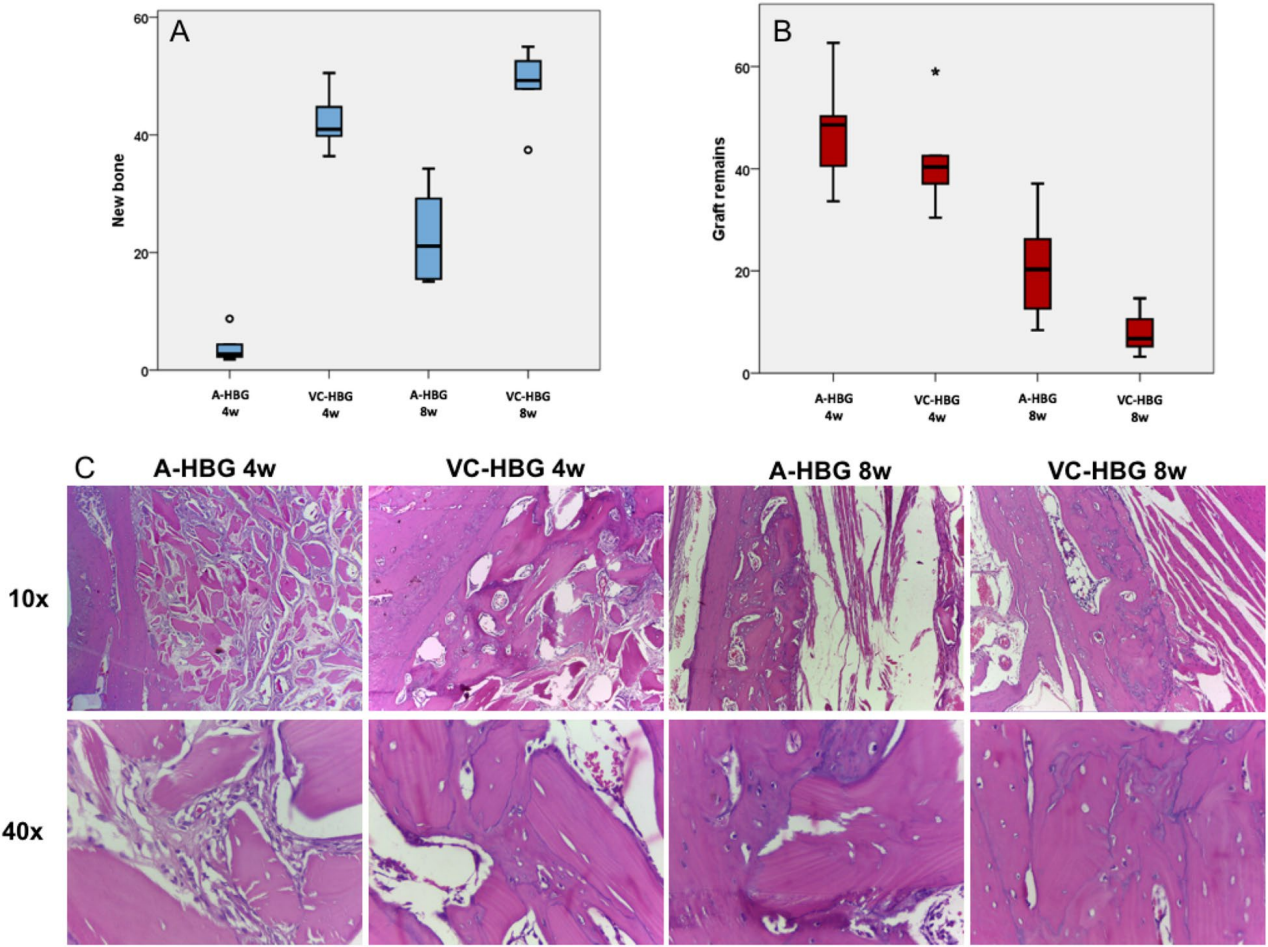
Association between new bone formation (**A**), graft remains (**B**) and graft type at each time point. Representative H&E pictures are depicted in (**C**) at 10× and 40× magnification. *A-HBG* acellular-human bone graft, *VC-HBG* viable cryopreserved-human bone graft.

The percentage of newly formed bone within the grafted area was significantly higher in the VC-HBG group at both time intervals (*p* = 0.001), see Fig. 2A and C.

#### Graft remains

There was no statistical difference between the VC-HBG and A-HBG group at 4-weeks for percentage of remaining graft particles (*p* = 0.047). At 8-weeks, the VC-HBG group showed a significantly lower percentage of graft remnants than the A-HBG group (8.07 ± 4.55% vs. 20.93 ± 11.37%, respectively; *p* = 0.047), see Fig. 2B and C.

### Immunohistochemical analyses

**Figure 3:**
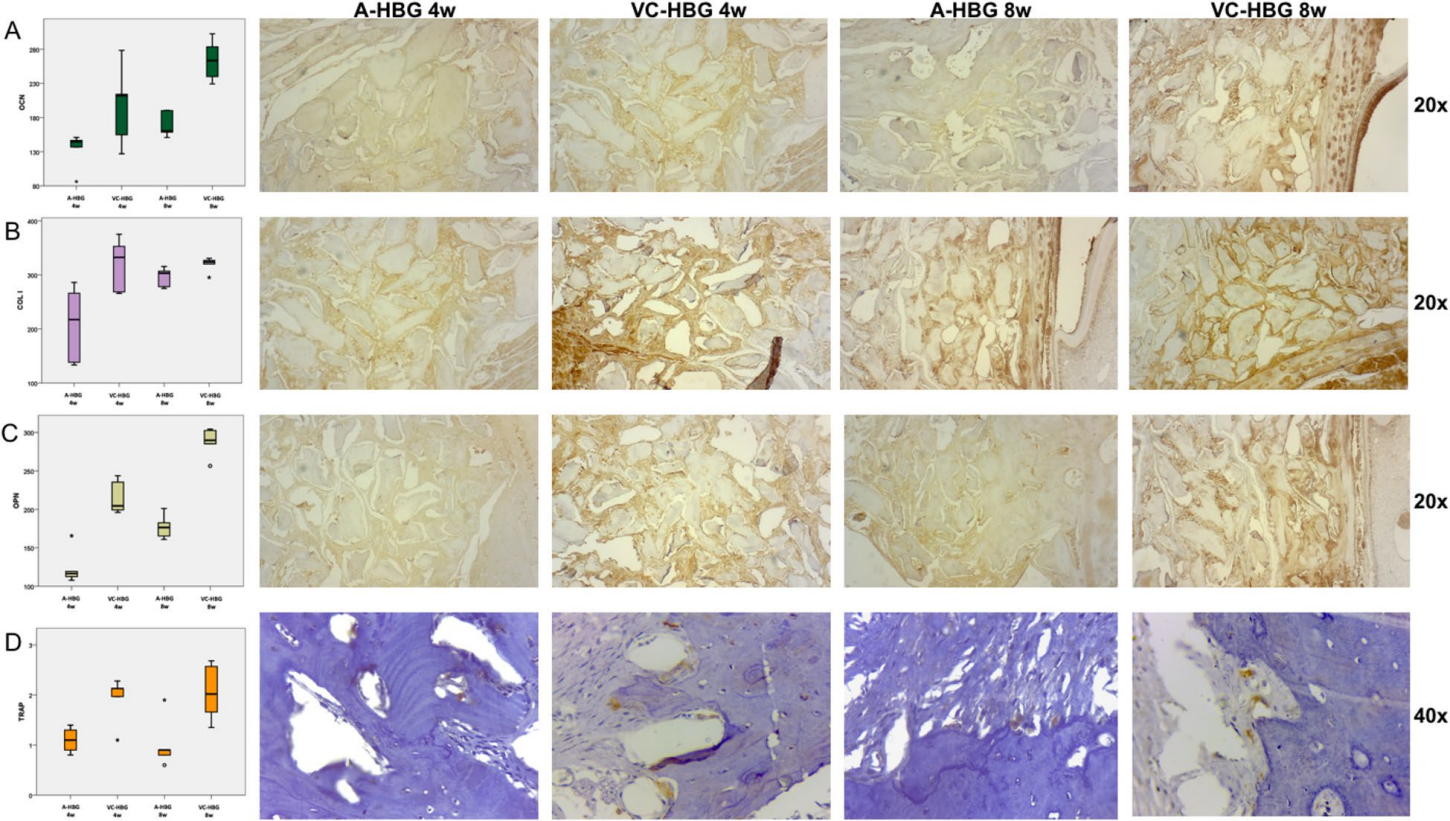
Association between OCN (**A**), COL1 (**B**), OPN (**C**), TRAP (**D**) and graft type at each time point. *A-HBG* acellular-human bone graft, *VC-HBG* viable cryopreserved-human bone graft, *OCN* osteocalcin, *COL1* collagen type 1, *OPN* osteopontin, *TRAP* tartrate-resistant acid phosphatase.

Quantitative immunohistochemical variables are presented through descriptive mean, median, standard deviation, minimum, maximum and interquartile range by type of graft in Table 3. Osteocalcin (OCN) expression was significantly higher (*p* < 0.001) in the VC-HBG group at 8-weeks (230.49 ± 56.34 pixels, px) compared to the A-HBG group (151.59 ± 29.23 px), see Fig. 3A. Overall, grafts at 8-weeks (216.95 ± 54.78 px) showed significantly higher OCN expression (*p* = 0.006) than those at 4 weeks (165.14 ± 54.72 px), see Fig. 3A. At both time points, Collagen I (COL 1) expression was significantly higher in the VC-HBG group (*p* = 0.021 at 4 weeks and *p* = 0.050 at 8 weeks), see Fig. 3B. Osteopontin (OPN) expression was significantly higher (*p* < 0.001) in the VC-HBG group at both time intervals (*p* < 0.001 for both 4 weeks and 8 weeks time points), see Fig. 3C. Tartrate-resistant acid phosphatase (TRAP) expression was significantly higher in the VC-HBG group at both time points. At 4-weeks, TRAP expression in the A-HBG group was 1.10 ± 0.25 cells/field of view (FOV) and 1.92 ± 0.47 cells/FOV in the VC-HBG group was (*p* = 0.009). At 8-weeks, TRAP expression for the VC-HBG group (2.06 ± 0.57 cells/FOV) was significantly higher (*p* = 0.016) than the A-HBG group (1.02 ± 0.51 cells/FOV), see Fig. 3D.

**Table 3:** Descriptive analysis of immunohistochemical quantitative variables by type of graft.

	OCN		COL I		OPN		TRAP	
	A-HBG (px)	VC-HBG (px)	A-HBG (px)	VC-HBG (px)	A-HBG (px)	VC-HBG (px)	A-HBG (cell/FOV)	VC-HBG (cell/FOV)
N	10	10	10	10	10	10	10	10
Mean	151.59	230.49	252.09	319.20	150.86	251.73	1.06	1.99
Median	151.03	234.59	276.75	325.49	163.19	250.06	0.90	2.08
Standard deviation	29.23	56.34	67.07	34.40	33.70	42.64	0.38	0.50
Minimum	86.29	126.95	133.35	265.69	107.79	195.91	0.60	1.10
Maximum	190.56	302.72	315.73	375.25	201.16	304.23	1.90	2.68
Interquartile range	25.09	81.93	106.54	48.92	62.51	89.79	0.53	0.77

*A-HBG* acellular-human bone graft, *FOV* field of view, *px* pixels, *VC-HBG* viable cryopreserved-human bone graft.

## Discussion

Bone augmentation of the jaws is often needed before dental implant placement procedures. Despite the development of several biomaterials and different techniques, the process of incorporation takes time and frequently results in loss of the initial grafted bone volume^11,12^. In our study, it was observed that animals which have been grafted with viable cryopreserved human bone chips (VC-HBG) showed a significantly greater increase in bone volume than animals that received A-HBG, at both 4- and 8-weeks time intervals.

Bone mineral density (BMD) has significantly changed in the control group while maintaining stability in the test group. Although the VC-HBG grafts showed lower scores compared to the A-HBG at 4-weeks interval, the A-HBG group BMD decreased significantly at 8-weeks and presented lower density than the VC-HBG group. As bone grafts are incorporated into the recipient site by creeping replacement, it is expected that the resorption of these grafts happen followed by osteoid deposition and later mineralization^13,14^. Thus, we hypothesize that the viable cell content of the VC-HBG group may have contributed to a more rapid bone turnover, decreasing the amount of mineral material by rising osteoclastic activity and, consequently, decreasing and stabilizing BMD. For the A-HBG group, the higher BMD at 4 weeks could be explained by slower graft resorption and therefore a higher mineral content. Even though it has not been directly tested, the immunohistochemistry data on TRAP cell count (showing more osteoclast activity in the VC-HBG group) and the quantity of graft remains at histomorphometry (more graft remains in the control group) corroborates with this hypothesis. Most studies comparing different types of bone scaffolds seeded with cells from different origins found the bone surface/volume ratio (BS/VR) higher in the cell groups than in the control groups^15–17^. On the other hand, the data from the present study showed that at 4 weeks the A-HBG group exhibited significantly higher BS/VR than the VC-HBG group and no statistical difference between groups at 8 weeks. A similar process that was found for the BMD might have resulted in a decrease of the bone surface, lowering the BS/VR at 4 weeks and achieving comparable ratios for both groups at 8 weeks. Through this perspective, the main advantage of the viable cellular content would be to speed up the graft substitution and bone maturation process, even though over time there would be no difference between the two groups. This feature could be convenient when translated to clinical settings lowering the healing time waited before placing dental implants. It is important to emphasize that the micro-CT assessment should be analyzed in conjunction with histology, histomorphometry and immunohistochemistry in order to interpret the results. Thus, higher BMD and BS/VR scores observed in the A-HBG group can be correlated with the histology sections where the high amount of graft remnants was observed at 4 weeks^18^.

Histomorphometrically, the VC-HBG group presented higher values of newly formed bone in both time intervals, which is consistent with our micro-CT results. In our previous study^10^, the percentage of newly formed bone in the cellularized group was higher than the acellular group at 4 weeks, however at 8 weeks it showed no statistical difference. In the present study, the VC-HBG higher new bone formation was observed at 8 weeks compared to the A-HBG group. Chamieh et al.^15^ also reported that the percentage of newly formed bone was significantly higher at days 14, 21, 28 and 35 post-grafting when using scaffolds seeded with MSCs from rat dental pulp compared with acellular scaffolds and with non-grafted defects. Wofford et al.^19^ also demonstrate greater bone formation in maxillary defects of mice treated with Gelfoam® and human MSCs from adipose tissue, compared to a group treated with Gelfoam® alone in a 4 weeks period. At 12 weeks, they did not find statistical difference, suggesting increased and early bone healing in the presence of human adipose tissue MSCs. Differently from what we found in our previous study^10^, there was no statistical difference in the percentage of graft remaining particles between the groups at 4 weeks. In this time interval, both groups had a greater amount of graft remnants than at 8 weeks, which is expected, since over time the graft particles should be replaced by new bone. At 8 weeks, the VC-HBG group had a significantly lower percentage of graft remnants than the A-HBG group. This result contributed to the understanding of the comparison of the parameters of BMD, BS/VS and percentage of bone remains: the test group at 8 weeks, despite having fewer remaining particles, presented greater bone volume and greater BMD, showing that there was greater bone formation in this group compared to the control at 8 weeks.

Type I collagen is expressed mainly by osteoblasts and represents an important organic component of the alveolar bone matrix^20^, having a critical role in the structure and function of bone tissue^21^. Type I collagen expression can be considered as a marker of early bone formation^22^. In our study, the COL I expression was higher in the VC-HBG than in the A-HBG group, at both time points. The greatest difference was observed at 4 weeks, showing that the presence of cells in the grafts contributed to a rapid increase in bone extracellular matrix deposition. This finding is consistent with the findings of Chamieh et al.^15^, who used the in situ hybridization technique, using a specific probe for Col1a1, and found strong collagen expression in scaffolds seeded with MSCs from rat dental pulp, while only weak signals associated with Col1a1 were observed in acellular scaffolds. Osteocalcin (OCN) is considered to be one of the most abundant non-collagenous proteins, as it is deposited in significant amounts in the bone matrix. It is predominantly synthesized and secreted by mature osteoblasts, hypertrophied chondrocytes and odontoblasts, has an affinity for calcium, and plays an important role in bone neoformation and mineralization^23,24^. The exact role of this protein in bone remodeling has not been fully elucidated, although its structure indicates interaction with calcium and hydroxyapatite crystals. However, it appears to be an important pathway for activating bone formation, due to its effect on osteoblasts. The appearance and increase in osteocalcin production coincides with the beginning of the mineralization process^25,26^. In our study, no significant difference in OCN expression was found at 4 weeks. At 8 weeks, the VC-HBG group showed a higher expression of OCN. This result differed from what was found in our previous study^10^, which showed a greater number of OCN-positive cells in the VC-HBG group in the 4-week interval and no statistical difference at 8 weeks. As OCN expression is a marker of late bone formation involved during the mineralization process^22–25^, this could be an explanation for the higher expression observed only at 8 weeks. A similar observation was made by Tera et al.^27^ in a study with ovariectomized rats, which received autogenous bone graft. OCN staining was not observed during initial crystal formation, but it could be found in the later stages of bone formation, with positivity of osteoblasts and newly formed bone matrix on day 45 and day 60, revealing characteristics of mature bone. De Ponte et al.^28^ also observed immunopositive cells for OCN only after 6 months of grafting the maxillary sinus elevation with fresh frozen acellular bone in humans. In bone, osteopontin (OPN) is synthesized and secreted by osteoblasts, osteocytes and osteoclasts^29^. It is a multifunctional matrix protein that is involved in the regulation of physiological and pathological mineralization. OPN serves both to unite the bone cells to the matrix, and to generate intracellular signals essential for the normal motility of osteoclasts in the bone^30^. It is produced by osteoblastic cells at different stages, and it has been suggested that its expression occurs in two peaks^31^. Therefore, this protein is detectable in bone marrow stem cells with intermediate levels of expression in the first stages of differentiation during the proliferation of cell precursors such as pre-osteoblasts and at high levels in osteoblasts^32^. In the present study, OPN expression was higher in the VC-HBG than in the A-HBG group, at both time intervals. Wofford et al.^19^ demonstrated remarkable expression of OPN in mice at week 4 with samples treated with Gelfoam® plus human MSCs derived from adipose tissue showing a more organized morphological distribution of this protein compared to the chaotic pattern in Gelfoam® samples without cells. Our study showed higher expression of proteins involved in osteogenesis (OCN, OPN and COL I) in the VC-HBG group compared to the A-HBG group, suggesting that there is an increase in the process of mineralization and bone formation in bone grafts with cells, corroborating with our previous study^10^.

Tartrate-resistant acid phosphatase is an enzyme found in osteoclasts and erythrocytes and is released during bone resorption, promoting degradation of the organic matrix. It is considered an important marker of osteoclastic activity^33^. In the present work, the VC-HBG group showed higher TRAP expression at both time points, unlike our previous findings with cell seeded graft^10^, in which there was greater positivity for TRAP only at 4 weeks. The detection of TRAP-positive osteoclasts, which were also found on the surfaces of newly formed bones, indicates an early process of ongoing remodeling, greater in the group with VC-HBG, which in this study remained until the time interval of 8 weeks.

This is the first study demonstrating the superior benefits of viable cryopreserved human bone grafts (VC-HBG) for mandibular augmentation. However, the animal model used imposes methodological limitations. Athymic rats tolerated the implantation of human cells and bone fragments well, without presenting adverse or immunological reactions. Nevertheless, the grafted material needs to be considered as a xenogeneic graft in this study, differently from what we would expect in the context of a human clinical use. In addition, it was not possible to determine precisely the chronological evolution of the incorporation and remodeling processes in the same individual due to the necessity of the use of different animals to assess the parameters in different time points. At last, we were not able to test the superiority of the VC-HBG for its final purpose, which is the placement of dental implants. Thus there is a need for future research on the behavior of the healed grafts in the presence of implants. Despite these limitations, the outcomes of this experimental research demonstrated that the VC-HBG has positive osteogenic properties, greater bone formation, higher rate of bone remodeling and a better overall incorporation in rats' mandibles compared to the A-HBG. Future studies in larger animals, with subsequent placement of dental implants, and clinical studies in humans are needed to assess the feasibility and long term outcomes resulting from the use of VC-HBG in the dental field.

## Methods

### Preparation of the viable cryopreserved-human bone graft (VC-HBG)

Fragments of fresh human vertebrae from the University of Miami Tissue Bank were harvested from cadaveric donors and ground in a manual bone crusher to produce bone chips of visually uniform sizes. Bone chips were then washed with Dulbecco's Phosphate Buffered Saline (DPBS), immersed in 10% dimethyl sulfoxide (DMSO) and deep-frozen at − 80 °C. VC-HBG were washed with DPBS after thawing before use.

### Preparation of the acellular-human bone graft (A-HBG)

Fragments of fresh human vertebrae from the University of Miami Tissue Bank were harvested from cadaveric donors and ground in a manual bone crusher to produce bone chips of visually uniform sizes. Bone chips were then extensively washed with Dulbecco's Phosphate Buffered Saline (DPBS), and freeze-dried to remove any viable cell content. At the time of use, VC-HBG were washed with DPBS after thawing. A-HBG were hydrated with DPBS before use. The macromorphological consistency, texture and appearance of both materials after preparation were indistinguishable. Particle size for both groups was between 250 and 1000 µm.

### Surgical procedure

This study was approved by the Institutional Animal Care and Use Committee (IACUC) at the University of Miami and all methods were performed in accordance with relevant regulations, including ARRIVE guidelines. Twenty athymic nude rats (NTac: NIH-Foxn1^rnu^, female, 10 weeks of age, weighing 150–200 g) were used in this study. Power analysis and sample size calculations were performed using G*Power 3.0 software. Sample power analysis using a One-Way ANOVA experimental design including 4 independent groups with α = 0.05, an effect of 0.85 and a sample size of 20 animals (for all groups) results in a power of > 0.80. The animals were randomly allocated into 2 different groups with 10 individuals, with 2 time intervals each (n = 5 animals per 'time point'). All surgeries were conducted by the same experienced oral surgeon (Daniel Deluiz) as previously described^10^. The rats were anesthetized with isoflurane and then placed on a 37 °C heating pad. Puralube® Vet Ointment was applied to the eyes to avoid drying. Antisepsis was made on the submandibular area with topical polyvinylpyrrolidone iodine. The submandibular surgical approach was performed through a linear incision involving the cutaneous and subcutaneous layers exposing the masseter muscle. The muscle was incised along the submandibular border taking care not to injure the facial nerve. A flap including the muscle and periosteum was raised exposing the lateral aspect of the rat’s mandible, thus creating a pouch underneath the masseter. The host bed was kept intact to avoid fracture or insufficient bone to stabilize the titanium screw. One 4.0 mm long, 1.5 mm diameter titanium microscrew (KLS Martin, Tuttlingen, Germany) was fixed on the lateral side of the mandible in order to maintain the space and to stabilize the graft using the tent-pole technique^34^. The material (VC-HBG or A-HBG) was then placed around and on top of the screw. The amount of material (0.1 cc) was chosen to completely fill the void between the bone and the elevated muscle and was then covered with a resorbable amniotic membrane in order to maintain material stability in place. Previous experimental surgeries were performed in animals not included in the study to define the strategy and standardization of the material application. Wound closure was made on the muscle and cutaneous planes with Vicryl 5–0 sutures. The mean duration of the procedure and anesthesia was 15 min for each animal. Antibiotic therapy was carried out with a single dose of Gentamicin (5.0 mg/kg IM), and buprenorphine (0.1 mg/kg SC) was administered once before surgery and 3 days after for pain control. The rats received a postoperatively soft diet for 3 days and then were returned to the standard dry food regimen. Water was provided ad libitum.

### Micro-CT scan analysis

The animals were euthanized at 4 and 8 weeks using CO_2_ inhalation and decapitated prior to scanning on the micro-CT device (SkyScan 1176, Bruker, Kontich, Belgium). Each head/sample was assigned a reference number to blind the examiner to the analyses. The imaging parameters were: 1 mm aluminum filter, exposure of 71 ms, 65 kV, 385 µA, 18 µm pixel size and 0.70° of rotation step. The images were reconstructed using the NRecon software (Bruker) with 30% of beam hardening correction. The analyses were performed in a blinded manner by the same investigator presenting an intra-examiner reproducibility Kappa index of 0.90. All datasets were aligned to the same orientation (lateral side of the mandible aligned horizontally and titanium screw aligned vertically) using the DataViewer software (Bruker). A region-of-interest (ROI1) comprising the entire grafted area (homogeneous calcified formation around the titanium screw plus spread microparticles resulting from excess material) and excluding the rat's mandible (and the titanium screw) was selected on the reconstructed images using the CTAnalyser software (Bruker). To avoid the analysis of the excess material which was not participating in bone augmentation, a new region within the ROI1 was selected—ROI2. ROI2 was defined in a standardized manner for all samples using a threshold of Hounsfield Unit (between + 200 and + 2000 HU). This threshold included the visually engrafted area (homogeneous calcified formation around the titanium screw) and excluded the excess particles (and the screw) from the measurements. The parameters assessed in the ROI2 were: gained bone volume (BV), bone mineral density (BMD), percent bone volume (PBV), bone surface/volume ratio (BS/VR) and bone surface density (BSD).

### Histology technique

Immediately after the micro-CT scan analysis, the mandibles were surgically removed together with the surrounding tissue and fixed with 10% neutral buffered formalin for 1-week. The titanium screws were removed taking care not to damage the grafted areas. The samples were decalcified with Cal-Ex decalcifying solution (Fisher Scientific, Massachusetts, USA) for 3 days, rinsed in distilled water, dehydrated in alcohol, cleared in xylene and embedded in paraffin. Paraffin block samples were then sliced at a 3 µm thickness, and then stained with Hematoxylin and Eosin for histological and histomorphometrical evaluation. Images were acquired using a Nikon Eclipse 90i light microscope.

### Histomorphometric measurements

The histomorphometric evaluation was performed by a blinded single trained investigator using 4 pictures from each sample (sufficient to cover the entire grafted area) at 10× magnification using ImageJ (NIH) software. The investigator had been previously tested for intra-examiner reproducibility presenting a Kappa index of 0.80. Newly formed bone as well as unincorporated graft particles were visually identified in the pictures and marked with the software’s selection tool. Errors in the automatic selection were checked and corrected manually. The defined areas of new bone were then colored in blue, while areas of unincorporated graft remains were colored in red. The color labeling allowed the selections to be more easily distinguishable. The amount of each parameter was calculated as a percentage from the entire image surface area.

### Immunohistochemical analysis

Immunohistochemical reactions were performed using the following primary antibodies to assess bone formation and remodeling: anti-OCN (Osteocalcin, clone: ab10911, Millipore, Massachusetts, USA, 1:200 dilution), anti-OPN (Osteopontin, clone: Akm2A1, Santa Cruz Biotechnology, Inc, 1:100 dilution), anti-COL 1 (Collagen type 1, clone: ab90395, Abcam, Massachusetts, USA, 1:100 dilution) and anti-TRAP (tartrate resistant acid phosphatase, clone: EPR15556, Abcam, Massachusetts, USA, 1:200 dilution). Sections were initially deparaffinized in xylene, hydrated in decreasing concentrations of absolute alcohol, 90°, 70° and submitted to antigenic recovery using a buffered solution of sodium citrate 10 nM, pH6.0 in a water bath at 90 °C for 20 min. Inactivation of endogenous peroxidase was performed by immersion of the sections in 3% hydrogen peroxide for 10 min, followed by incubation with primary antibodies in a humid chamber, at room temperature for 60 min. Positive and negative controls (omission of primary antibodies) were included in all reactions, according to the manufacturers' guidelines. After incubation with the primary antibodies, the sections were incubated with the secondary antibodies using a biotin-free immunoenzymatic antigen detection system and revealed with a diaminobenzidine chromogen from the same system (Reveal Polyvalent HRP-DAB detection system, Cat# SPD-125, Spring Biosciences, California, United States). Samples were counterstained with hematoxylin. Using light microscopy, 10 fields of view (FOV) of each slide (covering the entire grafted area) were taken at 40× magnification to quantify the number of TRAP positive cells. The cells were counted within the area of each FOV and the mean count was calculated for the corresponding sample (each sample cell count is a result of the mean of its FOVs). The mean cell count for each group was compared between groups^12^. For the OCN, OPN and COL1 quantification, 4 pictures from each sample at 10× magnification were analyzed using the ImageJ software (NIH). The positive reaction area was selected with the “threshold color” tool and calculated in pixels by the software. The positivity threshold was visually determined in agreement by two different blinded evaluators and the same value range was used to assess all the samples.

### Statistical analysis

Statistical analysis was performed using SPSS (IBM analytics). The significance level was set at 5% (*p* < 0.05). Student's t-test of independent samples was performed to verify the difference in the variables studied between the two types of graft (A-HBG and VC-HBG) at 4 weeks and 8 weeks. Kolmogorov–Smirnov test was used to verify the non-rejection of the assumption of normality of the distribution, so a parametric test was used. Two-way analysis of variance (ANOVA) was used to evaluate the effect of the type of graft and the effect of time, in addition to the interaction between them, in all the variables studied. To verify the assumptions of the analysis, the distribution of standardized errors was evaluated using the Kolmogorov–Smirnov (with Lilliefors correction) and Shapiro–Wilk normality tests, in addition to the evaluation of the Normal Q–Q Chart. After analysis of residuals, there was no significant rejection of the assumption of normality of data distribution by any variable of the study, validating the parametric test used.

## Data Availability

The data that supports the findings of this study are available from the corresponding author upon request.
